# Whole-genome sequencing of three ciprofloxacin-resistant *Salmonella* Reading (ST93) strains, an emerging *Salmonella* serovar in the poultry sector of Pakistan

**DOI:** 10.1128/mra.00006-24

**Published:** 2024-08-20

**Authors:** Abubakar Siddique, Azka Tauqeer, Amjad Ali, Aitezaz Ahsan, Sajid Iqbal, Ami Patel, Terence Moore, Erika Ganda, Abdur Rahman

**Affiliations:** 1 Atta Ur Rahman School of Applied Biosciences (ASAB), National University of Sciences and Technology (NUST), Islamabad, Pakistan; 2 Department of Veterinary Medicine, Institute of Preventive Veterinary Sciences, College of Animal Sciences, Zhejiang University, Hangzhou, China; 3 Animal Sciences Institute, NARC, Islamabad, Pakistan; 4 Maryland Department of Health and Laboratories Administration, Baltimore, Maryland, USA; 5 Department of Animal Sciences and Nutrition, Pennsylvania State University, University Park, Pennsylvania, USA; University of Maryland School of Medicine, Baltimore, Maryland, USA

**Keywords:** poultry meat, *Salmonella* Reading, *Salmonella enterica*, WGS, antimicrobial resistance

## Abstract

In this study, we performed whole-genome sequencing of three ciprofloxacin-resistant *Salmonella* Reading strains isolated from poultry meat. Genomes of *S*. Reading strains contained an average of 4.81 Mbp size with 52.1% GC. The isolates exhibited *bla_OXA-10_*, *aac* [*6′*]*-Iaa*, *aadA1*, *cmlA1*, *qnrS1*, and *tetA* resistance genes and *IncX1* and *IncX2* plasmids.

## ANNOUNCEMENT

*Salmonella* Reading is a rare serotype of *Salmonella enterica* subsp. *enterica*, identified in various animal hosts, including poultry. An outbreak of *S*. Reading from food items was previously reported in Canada from 2014 to 2015, with 31 confirmed cases involving people of Mediterranean descent (1). The frequent exposure of *Salmonella* to antimicrobials, including those used for therapeutics or as a growth promotor in poultry farms, has led to the emergence of antimicrobial-resistant serotypes (2, 3).

*Salmonella* strains were isolated from chicken meat collected from retail market stores in the Lahore and Rahim Yar Khan districts of Pakistan. Sliced tissues were pre-enriched in 1:9 buffered peptone water (BPW) (Oxoid, UK) and incubated at 37°C for 8 hours. Then, 0.1 mL of BPW suspension was added to 10 mL of Selenite F-broth (HiMedia, India) and incubated for 24 hours at 37°C. A loop of broth culture was streaked over *Salmonella-Shigella* (SS) agar (Oxoid, UK) and incubated at 37°C for 24 hours. PCR was used to confirm the *S. enteri*ca species identification through the *invA* gene. The phenotypic characteristics and antibiotic susceptibility of *S*. Reading strains isolated from poultry meat have been evaluated through CLSI guidelines in our previous study (4). Pure colonies were cultured overnight on SS agar at 37°C for genomic DNA extraction. DNA extraction was performed using the Roche MagNA Pure 24 platform. Library preparation was performed using Illumina DNA prep, and sequencing was performed through the Illumina MiSeq instrument with 2 × 150 bp paired-end reads, according to standard Illumina protocols. FastQC v0.11.9 and Trimmomatic v0.39 were used to preprocess the raw reads (5). Genome sequences were assembled using SPAdes v3.2. NCBI Prokaryotic Genome Annotation Pipeline was used to annotate the genomes. SISTR v1.0 was used to serotype the isolates, and the sequence types were determined using MLST 2.0 (6). Resistome was identified using the ResFinder 3.2 and CARD database, while the plasmids and *Salmonella* pathogenicity islands were determined by Plasmid Finder 2.1 and SPIFinder 1.0, respectively (7). The SNP-based phylogenetic analysis was performed using CSI phylogeny 1.4 (https://cge.cbs.dtu.dk/services/CSIPhylogeny/). *S*. Reading strain (SAMN14504941) was used as a reference strain (8).

**Fig 1:**
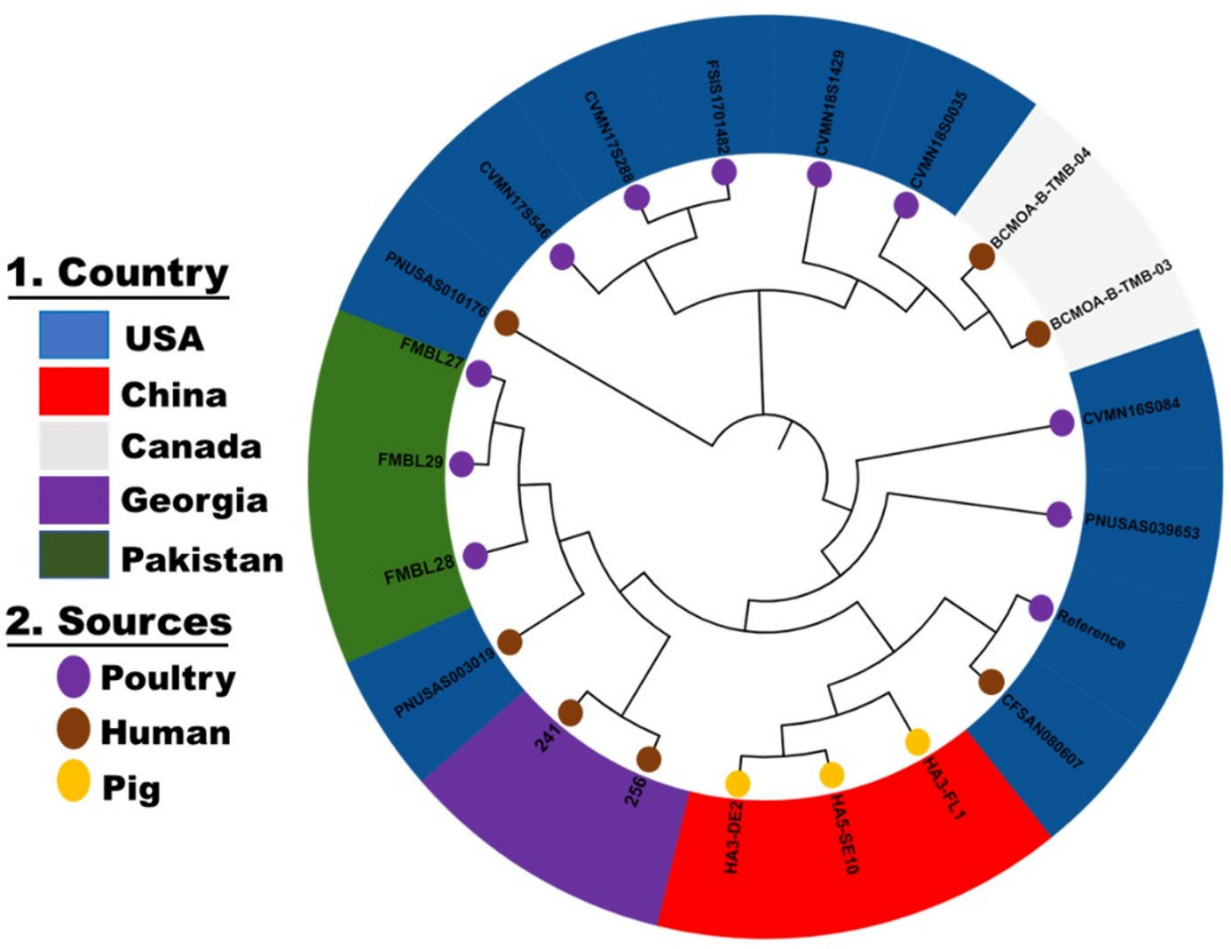
SNP-based phylogenetic tree composed of three *Salmonella* Reading and an additional 18 *Salmonella* Reading strains from different sources and countries downloaded from NCBI. This figure was generated with iTOL v.5.5 (https://itol.embl.de).

The genomic features of *Salmonella* isolates FMBL 27, FMBL 28, and FMBL 29 are presented in Table 1. The average genome size of strains was 4.83 ± 0.04 Mb, which is in agreement with the previous study (1). All the *Salmonella* isolates were serotyped as *Salmonella* Reading. The strains were identified as sequence type (ST93). We identified different antimicrobial resistance genes *aac* [6′]-*Iaa*, *aadA1*, *bla _OXA-10_*, *qnrS1*, *floR*, and *tetA*. The nonsynonymous point mutations in *gyrA* and *parC* were also identified. Among *S*. Reading isolates, two plasmid replicons, *IncX1* (41.2 kb) and *IncX2* (34.2 kb) were identified (Table 1). Several *Salmonella* pathogenicity islands, including SPI-1, SPI-2, SPI-3, SPI-4, SPI-5, SPI-8, SPI-9, SP-13, SP-14, and C63PI, were also identified in the genomes. According to an SNP-based phylogenetic tree, our strains were closely related to each other, and they formed a clade with human isolates from the USA and Georgia (Fig. 1).

**TABLE 1:** Genomic characteristics, genotypes, and sequence types (STs) of *S. enterica* subsp. *enterica* serotype Reading isolates from chicken meat in Pakistan

Sequence ID	Laboratory ID	Isolation source	No. of reads	No. of contigs	Genome size (Mbp)	% GC	*N*_50_ (bp)	Genome coverage	Plasmid	Resistance genotype	Accession no.
FMBL27	PML1	Poultry meat	1,104,907	26	4.858	52.2	385,860	67	*IncX1,* *IncX2*	*fosA7, aac(6')-Iaa, aadA1, ampc1, qnrS1, floR, tet(A), blA**_OXA-10_***	ABAJLX000000000.1
FMBL28	PML2	Poultry meat	1,130,561	29	4.794	52.1	205,445	70	*IncX2*	*fosA7, aac(6')-Iaa, qnrS1, floR, tet(A*)	ABAJLY000000000.1
FMBL29	PML3	Poultry meat	1,301,516	26	4.853	52.2	216,067	79	*IncX2*	*aac(6')-Iaa,* *blA**_OXA-10_,** qnrS1, floR, tet(A), cmlA1*	ABAJLK000000000.1

## Data Availability

The whole-genome sequencing project has been deposited in NCBI under accession no. listed in Table 1. The BioProject accession is PRJNA271470. The sequencing read numbers are SRX10773067, SRX10773069, and SRX10773146 for FMBL27, FMBL28, and FMBL29, respectively.
